# Application of the protection motivation theory for predicting COVID-19 preventive behaviors in Hormozgan, Iran: a cross-sectional study

**DOI:** 10.1186/s12889-021-10500-w

**Published:** 2021-03-08

**Authors:** Roghayeh Ezati Rad, Shokrollah Mohseni, Hesamaddin Kamalzadeh Takhti, Mehdi Hassani Azad, Nahid Shahabi, Teamur Aghamolaei, Fatemeh Norozian

**Affiliations:** 1 Student Research Committee, Hormozgan University of Medical Sciences, Bandar Abbas, Iran; 2 Social Determinants in Health Promotion Research Center, Hormozgan Health Institute, Hormozgan University of Medical Sciences, Bandar Abbas, Iran; 3 Department of Community Medicine, School of Medicine, Hormozgan University of Medical Sciences, Bandar Abbas, Iran; 4 Infectious and Tropical Diseases Research Center, Hormozgan Health Institute, Hormozgan University of Medical Sciences, Bandar Abbas, Iran; 5 Cardiovascular Research Center, Hormozgan University of Medical Sciences, Bandar Abbas, Iran

**Keywords:** Protection motivation theory, Behavior, COVID-19, Iran

## Abstract

**Background:**

The high prevalence and mortality of coronavirus disease 2019 (COVID-19) have made it the most important health and social challenge around the world. However, this disease can be largely prevented by adherence to hygienic principles and protective behaviors. It seems that identifying the processes involved in protective health behaviors can be effective in planning and implementing suitable interventions to encourage the community toward protective behaviors. Therefore, the present study aimed to predict the preventive behaviors of COVID-19 according to the Protection Motivation Theory (PMT).

**Methods:**

This cross-sectional study was conducted over 2 months in Hormozgan Province, Iran. The study population consisted of all citizens above the age of 15 years. An online questionnaire was used to collect the data. The questionnaire link was available to the participants through social networks. The questionnaire consisted of two sections, including the demographic information and the PMT constructs. All statistical calculations and hypothesis testing were performed in SPSS Version 21 and AMOS Version 21. The significance level was considered to be 0.05 for hypothesis testing.

**Results:**

A total of 2032 subjects, with the mean age of 34.84 ± 9.8 years (*r* = 15–98), participated in this study. Most of the participants were 31–40 years old, female (60.4%), married (72%), urban residents (87.3%), and employed (58.8%). The majority of them also had a bachelor’s degree or higher (58.8%). Significant positive correlations were observed between the preventive behaviors of COVID-19 and the perceived vulnerability (*r* = 0.192, *P* < 0.001), perceived severity (*r* = 0.092, *P* < 0.001), response efficacy (*r* = 0.398, *P* < 0.001), self-efficacy (*r* = 0.497, *P* < 0.001), and protection motivation (*r* = 0.595, *P* < 0.001). On the other hand, significant negative correlations were found between the preventive behaviors of COVID-19 and maladaptive behavior rewards (*r* = − 0.243, *P* < 0.001) and perceived costs (*r* = − 0.121, *P* < 0.001).

**Conclusion:**

The present findings showed that maladaptive behavior reward and fear negatively predicted the protective behaviors. On the other hand, response efficacy and self-efficacy positively predicted the protective behaviors; the impact of self-efficacy was the strongest. Overall, the information provided in this study can contribute to health policymaking in Iran.

## Background

Coronavirus disease 2019 (COVID-19), which was first detected in Wuhan, China, in December 2019, is a new respiratory disease, caused by a newly discovered coronavirus [1]. The World Health Organization (WHO) introduced COVID-19 as an international public health emergency on January 30, 2020 [2] and declared it as a pandemic on March 11, 2020 [3]. This disease has both short- and long-term impacts on communities, health systems, and individuals [4]. The uncertainties surrounding the nature of this global pandemic, social restrictions, preventive measures, and lockdown have caused physical and mental problems for individuals [5].

In addition to quarantine policies, social distancing [6, 7] and personal hygiene are considered essential by the WHO to combat COVID-19. Some preventive measures include social distancing (at least a one-meter distance), use of face masks, covering the face with a tissue paper or cloth while coughing or sneezing, washing the hands regularly, and not touching the mouth, nose, or eyes. Besides, stress and anxiety management can help prevent this disease [6, 8, 9]. In addition to these individual measures, it is important to take other protective measures into account, such as occupational health, employees’ physical health, proper ventilation in closed environments, and access to valid health information to control the disease [1, 10].

The hot weather of the summer, the impossibility of proper ventilation during this season, and social gatherings in closed environments led to the increased prevalence of COVID-19 during summer. Besides, high humidity made it difficult for people to use face masks outdoors during summer. In Iran, the cultural background of people, who are known to be hospitable, was also influential in the increased prevalence of this disease. Generally, hospitality is more prominent in southern provinces of Iran, especially in Hormozgan Province [11]. Also, close family and social relationships in this province could affect collective activities and norms during the COVID-19 epidemic [12, 13]. It should be noted that the religious beliefs of Iranians and Hormozgan residents may influence stress management and mental relaxation [14, 15] .

COVID-19 is a highly contagious disease, which can rapidly spread and infect many people. The adverse outcomes of this disease are severe and associated with acute respiratory problems and mortality. However, no definitive treatment or vaccine has been developed for this disease so far; therefore, protective measures seem to be essential [8, 16]. Generally, healthy behavior theories can help us identify the factors involved in protective behaviors to plan health promotion programs. The Protection Motivation Theory (PMT), which was first introduced by Rogers in 1975, has been widely used as a framework to predict protective behaviors [17, 18]. PMT assumes that adopting a protective behavior against health threats is dependent on personal motivation for self-protection.

In PMT, fear is appraised to predict and encourage protective behaviors and explain the cognitive processes involved in threat and coping appraisals [18]. Threat and coping appraisals can lead to adaptive or maladaptive responses, which are considered as threats to one’s health [19]. In PMT, threat appraisal depends on the following factors: (1) one’s belief in the severity of the problem (perceived severity); (2) one’s estimation of the chance of being affected by the disease (perceived vulnerability); and (3) one’s belief in the positive aspects of unhealthy behaviors (perceived rewards). Therefore, if the perceived severity and vulnerability are high, and the perceived rewards are low, there is a stronger motivation for engagement in health-promoting behaviors.

**Fig. 1:**
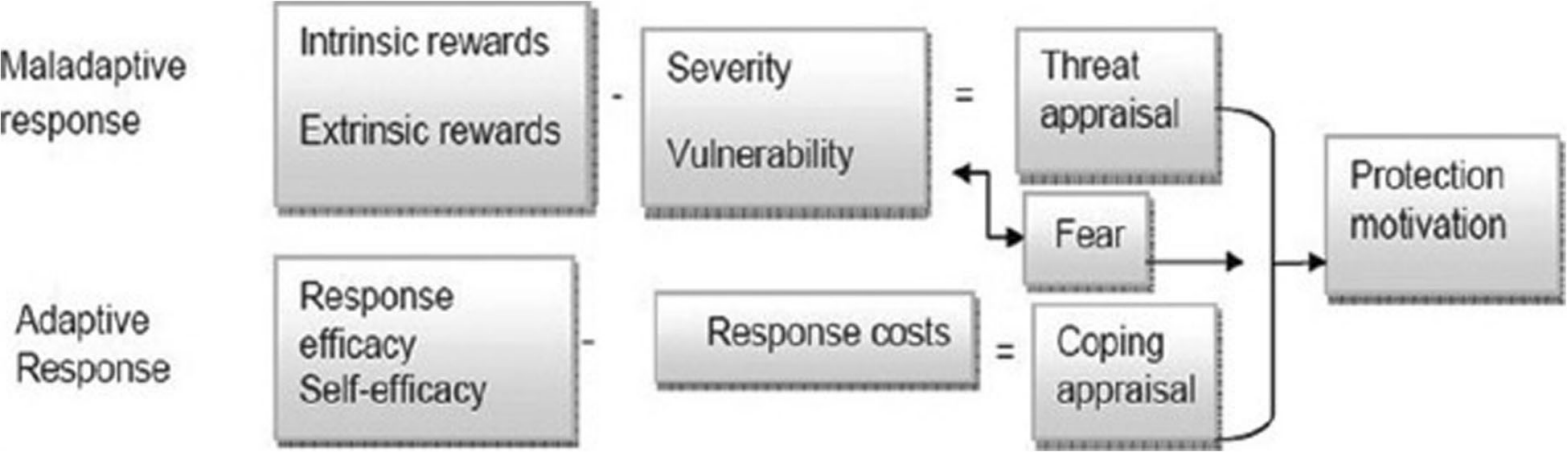
The framework of the Protection Motivation Theory (PMT)

In PMT, coping appraisal involves: (1) one’s evaluation of the efficacy of the protective behavior in coping with the threat (response efficacy); (2) one’s belief in one’s own capability of managing protective behaviors (self-efficacy); and (3) one’s estimation of costs (including money, time, and energy) and efforts to perform protective behaviors (perceived response cost). Overall, the response efficacy and self-efficacy are expected to reinforce coping appraisal, while the response cost is expected to reduce it (Fig. 1) [20].

Generally, fear is a mediator between perceived vulnerability, perceived severity, and threat appraisal. Therefore, if one feels vulnerable to a serious health threat, the level of fear is increased, and one is further motivated to adopt a preventive/protective behavior. During global pandemics, such as the COVID-19 pandemic, people experience fear and anxiety and realize that there is no definitive treatment for the disease. Also, fear of increased patient morbidity and mortality has raised public concerns and led to public panic, stress, and mental health problems [21, 22].

Coping and threat appraisal processes are integrated to develop protection motivation [23]. The literature suggests that PMT can accurately predict the adoption or non-adoption of protective behaviors [24–26]. So far, PMT has been used to investigate different behaviors, such as administration of influenza vaccine [27], preventive behaviors during the H1N1 pandemic [24], cancer preventive and sun-protection behaviors [28, 29], SARS preventive behaviors [30], and preventive behaviors for infectious diseases and skin cancer [31–33].

Iran is located in West Asia, and Hormozgan Province is located in south of Iran on the Persian Gulf coastline. This province consists of 13 counties, and its population is estimated at 177,641,500 according to the census in 2017 [34–36]. Since no definitive treatment or vaccine has been discovered for COVID-19, adopting protective behaviors is the best way to control this disease. Therefore, understanding the adaptive and maladaptive responses can help us identify the causes of people’s reluctance to use protective behaviors and the ways to address these issues. Therefore, this study was conducted to predict the protective behaviors of COVID-19, based on the PMT in the population of Hormozgan Province, Iran.

## Methods

### Study design

This cross-sectional study was conducted over 2 months (from March 2020 to April 2020) in Hormozgan Province, Iran. The questionnaire was available in the online Google Docs platform. The target population consisted of Hormozgan Province residents, aged above 15 years.

This research was approved by the Ethics Committee of Hormozgan University of Medical Sciences (ID No.: 980470, Ethics Code: IR.HUMS.REC.1398.479). The participants completed the questionnaires voluntarily, and their voluntary participation was appreciated on the first page of the questionnaire.

### Sampling method

Considering the risk of COVID-19 transmission through paper questionnaires, the data were collected online, using different messaging platforms (e.g., WhatsApp and Telegram channels of the research centers, School of Health, and Hormozgan University of Medical Sciences). The link to the questionnaire was sent in different online groups. The samples that received the link were asked to complete the questionnaire and send it to other people they know. Also, with the cooperation of health centers, the questionnaire link was sent to all individuals who were covered by the health centers in villages and cities. Besides, the link was sent to ten online information groups related to COVID-19 through the university’s public relations network.

In this study, considering the data collection method, there was no limitation in the number of samples. Also, completing the questionnaires was completely voluntary. On the first page of the questionnaire, after explaining the objectives of the study, the participants were asked to sign a consent form. The inclusion criteria were the minimum age of 15 years, literacy, and residence in Hormozgan Province. On the other hand, the exclusion criterion was unwillingness to participate in the study.

### Data collection

The data collection instrument was a researcher-made questionnaire. The validity of this questionnaire was assessed by the content validity method. The content of the questionnaire was extracted from credible sources, including relevant books and academic papers. It was also approved by a panel of three experts (health education and promotion specialists) after the necessary changes were made both qualitatively and quantitatively. The experts were asked to rate the items in terms of the words chosen, the order of presentation, and scoring. According to their feedback, revisions were made in the questionnaire. Its reliability was approved, based on the Cronbach’s alpha method. The questionnaire consisted of two parts. The first part included the demographic information, such as age, gender, marital status, education, occupation, background diseases, and history of smoking tobacco. The second part was related to the PMT constructs. All constructs of PMT, except for behavior, were rated on a five-point Likert scale (range: 1–5), ranging from “strongly agree” to “strongly disagree”. Also, the behavior construct was rated on a five-point scale (range: 0–4), ranging from “never” to “always”.

The perceived vulnerability (e.g., “I may also get infected with COVID-19.”) was tested in four items, with the scores ranging from 4 to 20. The perceived severity (e.g., “There is a chance of early death in the event of infection with COVID-19.”) was tested in six items, with the scores ranging from 6 to 30. Also, the perceived reward of maladaptive behaviors (e.g., “It is easier to breathe without a mask.”) was tested in seven items, with the scores ranging from 7 to 35. Besides, the perceived response efficacy (e.g., “Recurrent washing of the hands with water and soap for at least 20 seconds can protect me against COVID-19.”) was tested in seven items, with the scores ranging from 7 to 35.

The perceived self-efficacy (e.g., “I can adequately and appropriately disinfect contaminated or suspected objects and areas.”) was tested in six items, with the scores ranging from 6 to 30. Also, the perceived response cost (e.g., “Disinfecting contaminated objects and areas is time-consuming.”) was tested in seven items, with the scores ranging from 7 to 35. Besides, fear was tested in six items (e.g., “When I think about COVID-19, I feel anxious.”), with the scores ranging from 6 to 30. Also, protection motivation (e.g., “I have decided not to travel until the disease is eradicated.”) was tested in seven items, with the scores ranging from 7 to 35. Finally, behavior (e.g. “I avoid kissing or shaking hands with others.”) was tested in ten items, with the scores ranging from 0 to 40. The questionnaire also included Yes/No questions about the history of hypertension, diabetes, kidney disease, and cardiovascular diseases, and the participants were asked to answer these questions.

### Data analysis

The frequency, percentage, mean, and standard deviation (SD) indices were measured to describe the data. To test the study hypotheses and explore the correlations of COVID-19 preventive behaviors with the demographic characteristics, t-test and one-way ANOVA test were used. Besides, to evaluate the correlations between the PMT constructs and preventive behaviors, a multivariate linear regression analysis was performed. Also, to investigate the interactions of the PMT constructs and accept or reject the conceptual theory for COVID-19 preventive behaviors, path analysis and structural equation modeling (SEM) were performed. All statistical analyses and hypothesis tests were carried out in SPSS Version 21 and AMOS Version 21, and the significance level was set at 0.05.

## Results

### Study sample

In the present study, 2219 participants were included and completed the questionnaires online. Nonetheless, 3.9% (*n* = 87) of the questionnaires were incomplete and discarded. Finally, 2032 questionnaires were analyzed in this study. The average age of the participants was 34.84 ± 9.8 years, ranging from 15 to 98 years. As the results showed, the mean score of protective behaviors was significantly higher among participants who were above 15 years, female, and married. Also, the mean scores of participants with a bachelor’s degree or higher, healthcare staff, urban residents, non-smokers, and employed people were higher than others. The demographic information of the participants and the mean scores of protective behaviors are summarized in Table 1.

**Table 1:** The participants’ demographic information

Demographic information	Categories	Frequency N (%)	Mean score of COVID-19 preventive behaviors (SD)	***P***-value
Age (years)	15–20	132 (6.5)	31.70 (5.961)	<.001
	21–30	549 (27)	34.39 (5.331)	
	31–40	862 (42.4)	35.83 (4.109)	
	41–50	363 (17.9)	36 (4.218)	
	> 50	126 (6.2)	36.44 (3.928)	
Gender	Male	804 (39.6)	34.88 (4.748)	.005
	Female	1228 (60.4)	35.48 (4.742)	
Marital status	Single	510 (25.1)	34.04 (5.360)	<.001
	Married	1464 (72)	35.64 (4.367)	
	Divorced/widow	58 (2.9)		
Level of education	Elementary school	36 (1.8)	33.57 (5.320)	<.001
	High school	138 (6.8)		
	Diploma	442 (21.8)	34.21 (5.394)	
	Associate degree	222 (10.9)	34.74 (4.761)	
	Bachelor’s degree or higher	1194 (58.8)	35.96 (4.246)	
Occupation	Student	73 (3.6)	32.98 (5.586)	<.001
	University student	107 (5.3)		
	Employee	934 (46)	35.91 (4.416)	
	Self-employed	252 (12.4)	34.31 (5.219)	
	Unemployed	255 (12.5)		
	Others	411 (20.2)	35.87 (3.943)	
Medical staff	Yes	354 (17.4)	36.21 (4.704)	<.001
	No	1678 (82.6)	35.04 (4.739)	
Chronic diseases	Hypertension	154 (7.6)	36.06 (3.995)	0.025
	Diabetes	79 (3.9)	34.73 (4.830)	0.334
	Kidney disease	101 (5)	34.46 (4.916)	0.089
	Cardiovascular disease	71 (3.5)	35.30 (4.083)	0.921
Smoking	Yes	217 (10.7)	34.24 (4.781)	0.001
	No	1815 (89.3)	35.36 (4.736)	
Place of residence	Urban	1773 (87.3)	35.4 (4.593)	<.001
	Rural	259 (12.7)	34.16 (5.617)	

Table 2 presents the Cronbach’s alpha coefficients and the range of scores for the PMT constructs. The crude mean scores, adjusted mean scores, percentages, and Pearson’s correlation coefficients of the PMT constructs were measured in respect to the target protective behaviors. In the present study, significant positive correlations were found between the preventive behaviors of COVID-19 and the perceived vulnerability, perceived severity, response efficacy, self-efficacy, and protection motivation. On the other hand, significant negative correlations were found between the preventive behaviors of COVID-19 and maladaptive behavior rewards and perceived costs (Table 2).

**Table 2:** Bivariate correlations of the PMT constructs and the protective behaviors of COVID-19

Variables	Item number	α	Range of score	Crude mean (SD)	Adjusted mean (SD)	Mean percentage	1	2	3	4	5	6	7	8	9
Vulnerability	4	0.773	4–20	16.11(2.37)	4.03(.594)	80.570		0.321^a^	−0.180^a^	0.284^a^	0.172^a^	−0.009	0.086^a^	0.263^a^	0.192^a^
Severity	6	0.765	6–30	21.69(3.66)	3.62(.610)	72.306			−0.200^a^	0.198^a^	0.042	0.035	0.035	0.169^a^	0.092^a^
Rewards	7	0.793	7–35	15.29(4.89)	2.18(.699)	43.676				−0.321^a^	−0.215^a^	0.422^a^	0.104^a^	−0.285^a^	−0.243^a^
Response efficacy	7	0.789	7–35	29.77(3.66)	4.25(.523)	85.049					0.484^a^	−0.159^a^	0.044	0.509^a^	0.398^a^
Self-efficacy	6	0.782	6–30	25.31(3.45)	4.22(.576)	84.381						−0.188^a^	−0.021	0.599^a^	0.497^a^
Costs	7	0.751	7–35	21.88(4.41)	3.13(.630)	62.512							0.321^a^	−0.076^a^	−0.121^a^
Fear	6	0.917	6–30	21.20(5.45)	3.53(.908)	70.666								0.321^a^	−0.024
Motivation	7	0.896	7–35	32.17(3.50)	4.60(.500)	91.924									0.595^a^
COVID-19 preventive behaviors	10	0.850	0–40	35.24 (4.75)	3.52(.475)	88.101									

a Correlation is significant at the 0.01 level

**Fig. 2:**
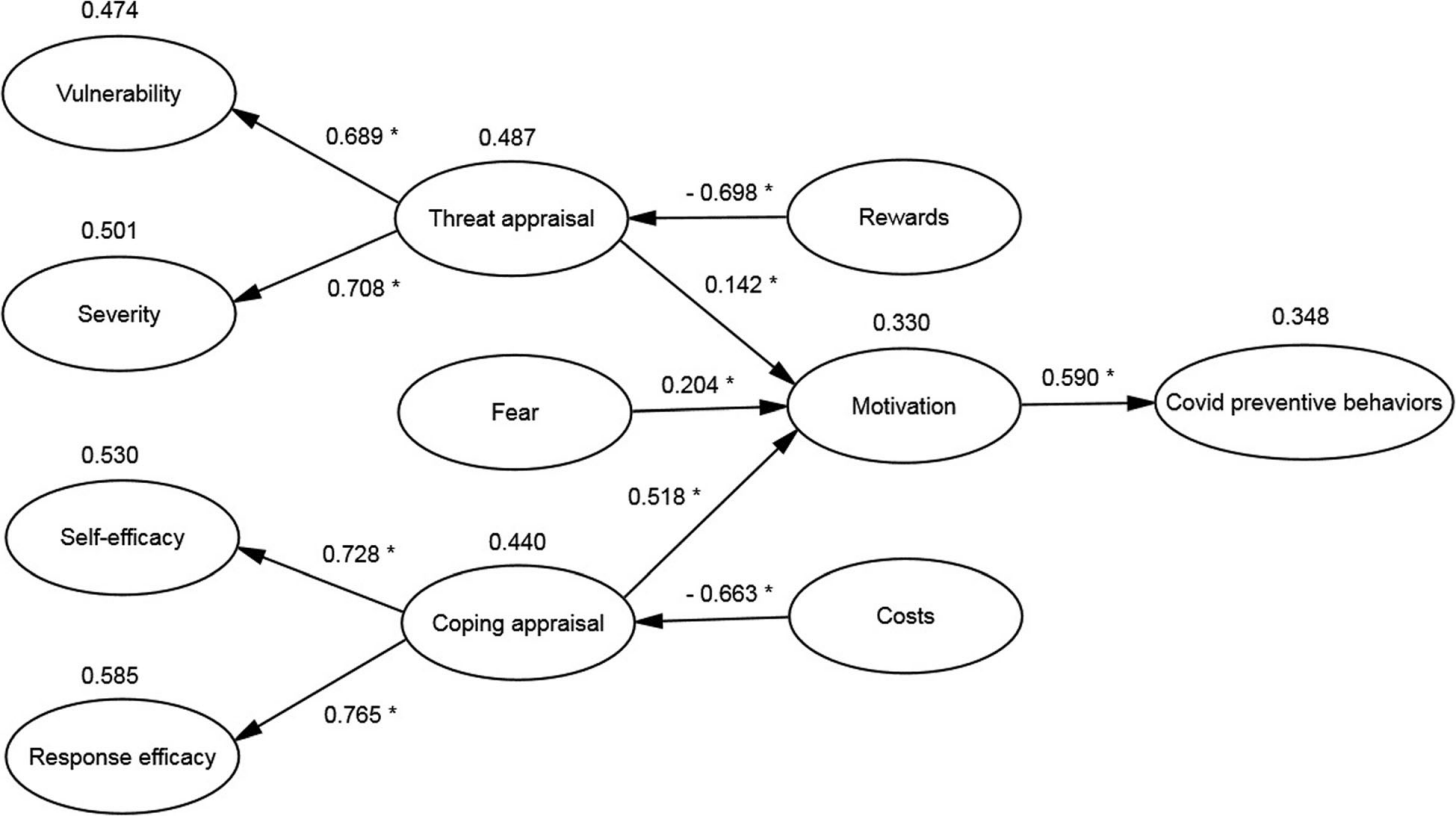
The structural equation modeling (SEM) of COVID-19 protective behaviors (*significant at 0.01)

Table 3 and Fig. 2 present the results of path analysis and SEM. Overall, the results of SEM showed that 35% of variance in the dependent variable (COVID-19 preventive behaviors) could be explained by the independent variable (motivation) (*R*^2^ = 0.348). The R^2^ for protection motivation was 0.330, which indicates that 33% of variance in the dependent variable (motivation) could be explained by the independent variables (coping appraisal, fear, and threat appraisal). Moreover, coping appraisal, threat appraisal, and fear were significantly correlated with protection motivation (*P* < 0.001). Besides, motivation protection was significantly correlated with COVID-19 preventive behaviors (*P* < 0.001) (Table 3 and Fig. 2).

**Table 3:** The path analysis of the PMT model of COVID-19 preventive behaviors (*n* = 2032)

Dependent variable	Independent variables	Path confidence	t-statistic	***R***^**2**^
COVID-19 preventive behaviors	Motivation	0.590	32.907	0.348
Motivation	Coping appraisal	0.518	28.538	0.330
	Threat appraisal	0.142	7.792	
	Fear	0.204	11.213

For the regression analysis of the PMT constructs, the backward regression method was applied. As shown in Table 4, maladaptive behavior rewards, response efficiency, self-efficacy, and fear predicted the protective behaviors of COVID-19. According to the regression analysis, the PMT constructs could explain 39% of variance in the protective behaviors of COVID-19. Among the PMT constructs, self-efficacy was the strongest predictor. Two variables, that is, reward and fear, were negatively correlated with the protective behaviors. In other words, higher scores of these two variables were associated with a lower score of COVID-19 preventive behaviors (Table 4).

**Table 4:** Prediction of COVID-19 preventive behaviors according to the multiple regression analysis (*n* = 2032)

	Unstandardized coefficients		Standardized coefficients	t	Sig.	95% Confidence interval for B		***R***^**2**^
	B	Std. error	Beta			Lower bound	Upper bound	
(Constant)	8.764	1.024		8.561	<.001	6.757	10.772	.398
Rewards	−.044	.018	−.046	−2.454	.014	−.080	−.009	
Response efficacy	.092	.028	.071	3.336	.001	.038	.146	
Self-efficacy	.253	.031	.184	8.176	<.001	.192	.313	
Fear	−.068	.015	−.078	−4.404	<.001	−.098	−.038	

The distribution (frequency and percentage) of protective behaviors, reported by the participants, is presented in Table 5. The results showed that 79.8% of the participants always avoided unnecessary travel; 78.6% always avoided kissing or hand-shaking; and 74.4% wore masks if they approached a suspected person (Table 5).

**Table 5:** Distribution of COVID-19 preventive behaviors in the participants

Preventive behaviors	Never N (%)	Seldom N (%)	Sometimes N (%)	Mostly N (%)	Always N (%)	Item mean	Item SD
Going out of the house only in emergencies	27 (1.3)	1 (0)	488 (24)	499 (24.6)	1017 (50)	3.22	0.902
Avoiding kissing or shaking hands	24 (1.2)	0 (0)	75 (3.7)	355 (16.5)	1598 (78.6)	3.71	0.648
Avoiding touching the mouth, nose, and eyes	10 (.5)	0 (0)	244 (12)	759 (37.4)	1019 (50)	3.37	0.729
Keeping a 1–2-m social distance	11 (.5)	2 (.1)	237 (11.7)	752 (37)	1030 (50.7)	3.37	0.734
Using tissues while coughing/sneezing	8 (.4)	0 (0)	130 (6.4)	446 (21.9)	1448 (71.3)	3.64	0.638
Avoiding crowded places	32 (1.6)	0 (0)	132 (6.5)	505 (24.9)	1363 (67.1)	3.56	0.752
Washing hands regularly with water and soap for at least 20 s	8 (.4)	1 (.0)	106 (5.2)	529 (26)	1388 (68.3)	3.62	0.625
Avoiding unnecessary travel	63 (3.1)	0 (0)	97 (4.8)	250 (12.3)	1622 (79.8)	3.66	0.833
Stress management	13 (.6)	0 (.0)	205 (10.1)	677 (33.3)	1137 (56)	3.44	0.725
Wearing masks while approaching suspected cases of COVID-19	11 (.5)	0 (.0)	141 (6.9)	369 (18.2)	1511 (74.4)	3.66	0.655

## Discussion

The present study aimed to predict the adoption of COVID-19 preventive behaviors, based on the PMT among Hormozgan residents, aged ≥15 years. The results revealed that maladaptive behavior rewards, response efficacy, self-efficacy, and fear predicted the protective behaviors. Among the PMT constructs, self-efficacy was the strongest predictor. Also, the mean score of COVID-19 preventive behaviors in the target population was above average, which can be due to awareness raising and management of the disease.

The present results showed a significant correlation between age and protective behaviors of COVID-19. In other words, older age was associated with a higher frequency of adopting protective behaviors, which could be attributed to the higher level of awareness and perceived threat at older age. Since older people are more prone to background diseases, they may be inclined to use protection more than others. Besides, the perceived severity, which is dependent on age, is higher among the elderly than the youth [37]. Moreover, in the present study, there was a significant association between preventive behaviors and education. In other words, individuals with a bachelor’s degree or higher adopted more protective behaviors, which can be due to their higher level of knowledge. Therefore, based on the present results, it is necessary to design interventions to raise the awareness of less educated people in Hormozgan Province.

The present results also showed that women adopted more preventive behaviors than men. Several studies on other ethnicities have also confirmed differences between males and females regarding their health beliefs and healthy behaviors. These results showed that certain gender-specific plans need to be designed to reinforce the preventive behaviors of COVID-19. Besides, the results of Pearson’s correlation coefficient test showed significant positive correlations between the preventive behaviors of COVID-19 and the perceived severity, vulnerability, response efficacy, self-efficacy, and protection motivation. On the other hand, significant negative correlations were found between the preventive behaviors of COVID-19 and the perceived rewards and costs; these findings are consistent with a number of previous studies [28, 38].

Considering the national and provincial restrictions for the management of COVID-19, which have led to limitations in many occupations, a large part of the community faces a significant economic burden. Therefore, it is necessary to pay attention to the possible adverse social and economic consequences of this disease, besides making attempts to control its spread [4, 39]. Moreover, economic vulnerability may result in people’s reluctance to engage in protective behaviors. It seems that planning and management of local authorities to reduce the economic burden (e.g., providing appropriate facilities in local health centers) can be effective in encouraging people to show protective behaviors [39].

The present findings also revealed that COVID-19 preventive behaviors were significantly and positively correlated with the perceived vulnerability. In other words, if people perceive themselves as vulnerable to the disease, they adopt more protective behaviors; this finding is consistent with the results reported by Babazadeh et al. [32] and Mohammadi et al. [40]. Furthermore, the present results showed that protective behaviors and perceived severity were positively and significantly correlated. Therefore, if people are made aware of the health consequences of COVID-19, they will adopt more protective behaviors. This finding is in agreement with the results reported by Tazval et al. [41], but inconsistent with the results reported by Zare et al. [42].

In the present study, a significant positive correlation was observed between the perceived vulnerability and the severity of COVID-19. This finding is consistent with the results of studies by Barati et al. [43], Zare et al. [28], and Park et al. [44]. According to their results, for a better understanding of the risks of this disease, a higher level of perceived vulnerability is needed; therefore, perception can strongly and positively affect the perceived threat of COVID-19. Also, a significant positive correlation was found between the perceived response efficacy of preventive behaviors and the individual’s self-efficacy and protection motivation. In other words, a greater understanding of the effectiveness of protective behaviors was followed by the increased level of self-efficacy, and vice versa. Moreover, a greater understanding of the effectiveness of protective behaviors was associated with higher motivation for protection, and vice versa; this finding is consistent with the study by Zare Sakhvidi et al. [28].

The present study also showed that COVID-19 preventive behaviors were significantly and positively correlated with protection motivation; however, this finding is inconsistent with the results reported by Kaviani et al. [45]. Based on the present results, the coping appraisal variables were the strongest predictors of protective behaviors in the target population. In line with several previous studies [28, 46], self-efficacy was the strongest predictor of these behaviors, followed by response efficacy. It seems that individuals with a higher level of self-efficacy perceive themselves to be capable of achievements; therefore, they show a tendency toward preventive behaviors of COVID-19.

Both response efficacy and self-efficacy are subsumed under coping appraisal in the PMT. The response efficacy refers to the effectiveness of coping responses in reducing threats. Generally, self-efficacy involves the individual’s perceived capability of showing a coping response. The regression coefficients showed that higher levels of self-efficacy and response efficacy could help increase motivation for preventing COVID-19. These findings are consistent with previous studies, which also adopted PMT as their theoretical framework.

The available coping strategies are effective in the adoption of protective behaviors. A high response efficacy strengthens self-protection and belief in the effectiveness of protective behaviors [47, 48]. The effect size of coping strategies or threats depends on the health issue to some extent. In the literature, the threat appraisal variables were the strongest predictors of cancer preventive behaviors. However, in terms of smoking, the coping appraisal variables were the strongest predictors of preventive behaviors [38]; the high educational level of the participants might be one of the contributing factors [49]. It should be noted that in the present study, the majority of the participants were in the age range of 31–40 years, which could be the main reason for the unprecedented effects of perceived severity and vulnerability in PMT on preventive behaviors. Overall, a higher self-efficacy can improve preventive behaviors in vulnerable populations [50, 51]. Also, health promotion programs, with the aim of improving self-efficacy in COVID-19 preventive behaviors, may improve preventive behaviors and promote social health.

In the current study, maladaptive behavior reward and fear were negatively correlated with preventive behaviors. In other words, higher levels of fear and maladaptive behavior reward were related to the lower probability of adopting protective behaviors. It seems that awareness of maladaptive behavior rewards is more important than the perceived cost of healthy behaviors. According to previous studies on the global outbreak of COVID-19, the psychological consequences of home quarantine and social restrictions include fear, anxiety, and depression, which can have negative impacts on protective behaviors [5].

According to the literature, governments need to find effective ways to disseminate unbiased information about COVID-19, train appropriate preventive strategies, ensure the availability of essential services and goods, and provide adequate financial support to overcome this crisis [1, 52]. Some other studies have reported similar findings regarding the use of condoms [53]. Conversely, in an investigation of cancer preventive behaviors in the workplace, the perceived costs were more important than the perceived rewards [28]. In the present study, protective behaviors were at a desirable level, which is consistent with a study by Barati et al. on COVID-19 protective behaviors in the hospital staff [37] and a study by Shahabi et al. on COVID-19 protective behaviors in Hormozgan Province [36]. Besides, a study by Wang et al. showed that the use of face masks during the COVID-19 pandemic could protect the physical and mental health of the public [6].

Since COVID-19 is a newly emerging disease, affecting many countries around the world, the information sources are being constantly updated. The adoption of acceptable protective behaviors is important during this pandemic. Overall, regular washing of the hands is the most important protective behavior, which was less adopted by the target population; therefore, awareness raising and education are of utmost importance. It is worth mentioning that behavioral change is generally a process that requires time. According to the theory adopted in this study, coping appraisal responses that lead to protection motivation are made after the threat appraisal process, because a threat needs to be identified before the appraisal of coping strategies. Therefore, attempts must be made as soon as possible to improve public awareness, create mutual trust, promote effective coping responses, and contribute to the achievement of plans.

### Strengths

The present study is one of the very few studies applying health promotion models to explore the preventive behaviors of COVID-19. This research was conducted on a large sample size within the shortest time possible.

### Limitations

The first limitation of this study is that online surveys can be only used by literate people, who have access to the Internet. Second, the majority of the participants in this study were young urban residents; therefore, the generalizability of the findings is limited. Third, physical health was reported by the participants themselves, and no acceptable evaluation tool was used. Fourth, people with mental illnesses were not included in this study, as they could not show proper preventive behaviors due to their psychological problems, such as stress and anxiety. Therefore, future studies are suggested to evaluate the performance of these individuals and propose appropriate plans.

## Conclusion

The present findings revealed that the rewards of maladaptive behaviors and fear were the negative predictors of the protective behaviors of COVID-19. It is generally difficult to control this disease in Hormozgan Province, considering the cultural background of people in this region (e.g., visiting family and friends, hand shaking, and cheek kissing) and its hot weather that causes individuals to stay indoors and avoid wearing masks. Therefore, implementation of appropriate interventional programs is necessary, with attention to the rewards of maladaptive behaviors. Also, to manage fear in the community, appropriate planning is necessary by sending appropriate health messages and preventing the spread of fake news. Moreover, using appropriate psychological techniques and coping behaviors is recommended. In this study, the response efficacy and self-efficacy were the positive predictors of protective behaviors (self-efficacy was the strongest). It seems that proper planning for interventions to increase self-efficacy in the community can be a positive step toward the prevention and control of this disease. In this study, the participants’ score of COVID-19 preventive behaviors was above average, which could be due to appropriate awareness raising and management of the disease in the province. Among preventive behaviors, avoidance of unnecessary travel, avoidance of kissing/shaking hands, and wearing masks were the most common behaviors. Overall, the present findings can be applied for policymaking in target populations.

## Data Availability

The datasets used and/or analyzed during the study are available from the corresponding author on reasonable request.

## Abbreviations

PMT: Protection Motivation Theory
WHO: World Health Organization
SARS: Severe Acute Respiratory Syndrome
H1N1: Influenza Swine Flu
COVID-19: Coronavirus Disease 2019
ANOVA: Analysis of Variance
SEM: Structural Equation Modeling
